# Some of the Rights and Duties of Dentists at Common Law

**Published:** 1893-08

**Authors:** Babson S. Ladd

**Affiliations:** Boston


### SOME OF THE RIGHTS AND DUTIES OF DENTISTS AT COMMON LAW.

BY BABSON S. LADD, BOSTON.

As preliminary to the view that I am to attempt to present some of the rights and duties of dentists at common law, I shall venture to remind you that in all employments demanding special skill and knowledge, be such employments classed as professional or mechanical, the general principles of law defining the civil responsibility and duties of persons thus employed, are the same. Taking, then, for illustration the list put by Dr. Elwell in his work on medical jurisprudence, it may be said that physicians, lawyers, engineers, machinists, ship-builders, and brokers, as well as all other classes of men whose employment is of the character I have indicated, are bound by the same general rules of law.

The law requires a dentist in his conduct towards his patient to exercise that reasonable degree of skill, coupled with learning and experience, which is ordinarily possessed by others of the dental profession.

States regulating the practice of dentistry simply operate to exclude from practice those persons (quacks and charlatans the courts have called them) who are incapable, from lack of the learning and experience ordinarily possessed by dental practitioners, of treating their patients with a reasonable degree of skill. All of this is for the protection of the public. That is common law.

Any one who assumes to be qualified for the exercise of any profession, art, or vocation is responsible for any damage that may result to those who employ him from the want of the necessary and proper knowledge, skill, and science which such profession demands.

He impliedly contracts with those who employ him that he has such skill and knowledge as will enable him properly to perform the duties of his calling. If he should be deficient in these respects, he violates his part of the contract, and must account in damages for any malpractice by which those who employ him sustain injury.

The failure of a course of treatment is not by any means conclusive of that want of professional skill by the practitioner. If his acts have been in accordance with the best known authority and skill, even though they should have been wrong, he will, in the eyes of the law, have done all that he could have done.

The standard of professional skill is never stationary. It must be kept up to and even with the constant advance of professional knowledge. Therefore the law demands *qualification* in the profession practised; not extraordinary skill such as belongs to only a few men of rare genius and endowments, but that degree which ordinarily characterizes the profession.

In keeping up with the march of the profession it is well to advance cautiously in experimental practice, for if the experiment in any particular case is rash and contrary to knowledge and usage of the profession, you will be liable for malpractice, as it matters not in such cases what your general skill may be.

Some standard by which to determine the propriety of treatment must be adopted, otherwise experiment will take the place of skill, and the restless experimenter the place of the educated, experienced practitioner. When, therefore, the treatment or operation is experimental, the practitioner will act at his peril, unless he first obtains the consent of the patient or some one in authority acting for the patient. In the wrongful act of a student or servant, the preceptor or employer would be wholly responsible for the evil results which might follow if performed while in his employment, the law holding that the omission is the omission of the principal. In regard to the claims of dentists for professional services, the law recognizes the difference in the value of time. In the absence of any agreement as to the amount to be paid for services rendered, the law recognizes an implied promise to pay so much as the dentist deserves to have, which amount will be governed by the scale of prices in general use among dentists of good standing.

On the other hand, he cannot exact compensation for services necessitated by his own lack of skill and care.

The patient, however, is not without some responsibility, and must faithfully comply with professional order or advice. Nothing can be clearer than the duty of the patient to co-operate.

In giving evidence in court the expert cannot be compelled to testify, and the only way to procure his testimony is to pay for it, the obligation to pay resting upon the party who calls the expert.

